# Prevalence of Anaemia among the Elderly in Malaysia and Its Associated Factors: Does Ethnicity Matter?

**DOI:** 10.1155/2018/1803025

**Published:** 2018-04-29

**Authors:** Muslimah Yusof, S. Maria Awaluddin, Maisarah Omar, Noor Ani Ahmad, Fazly Azry Abdul Aziz, Rasidah Jamaluddin, Tahir Aris, Maw Pin Tan

**Affiliations:** ^1^Institute for Public Health, National Institute of Health, Ministry of Health Malaysia, 50590 Jalan Bangsar, Kuala Lumpur, Malaysia; ^2^Division of Geriatric Medicine, Faculty of Medicine, University of Malaya, Kuala Lumpur, Malaysia

## Abstract

**Introduction:**

Anaemia is common among the elderly, yet it remains an underresearched clinical condition. This study investigates ethnic differences in prevalence of anaemia and identifies potential factors associated with anaemia in the elderly.

**Methods:**

Data from the National Health & Morbidity Survey (NHMS) 2015 conducted by Ministry of Health was analyzed. Haemoglobin levels were measured using point-of-care testing, HemoCue® Hb 201+ System©, from consenting individuals. Demographic information and other clinical information were obtained through a structured questionnaire. Descriptive and multivariate analyses were conducted and significant results were presented as adjusted odds ratio.

**Results:**

A total of 3794 participants aged 60 years and older responded to the anaemia module with a response rate of 93.7%. 64.0% of respondents were of Malay ethnicity, 21.6% were Chinese, 6.1% were Indians, and 8.3% were of other ethnicities. The overall prevalence of anaemia among older people was 35.3%. The highest prevalence of anaemia was found among respondents of Indian ethnicity (45.5%). The Indian (aOR: 1.72; 95% CI 1.26–2.34) and Malay (aOR: 1.25; 95% CI 1.04–1.49) ethnic groups were more likely to be anaemic in comparison to those of Chinese ethnicity. Anaemia in older people was also associated with increasing age, history of hospital admission, and the presence of diabetes mellitus.

**Conclusion:**

Anaemia in the elderly is associated with Indian and Malay ethnicities, increasing age, hospitalization, and diabetes. Our study has identified important information on a common condition which will guide and assist future studies in reducing the burden of anaemia.

## 1. Introduction

The World Health Organization defines anaemia as a haemoglobin level of less than 12 g per dL (120 g per L) in women and less than 13 g per dL (130 g per L) in men [1]. Anaemia was estimated to be present in one-third of the global population in 2010 [2]. Anaemia in older persons is likely to contribute significantly to the global burden of disease as it is independently associated with increased morbidity and mortality [3, 4]. Even mild anaemia can affect the quality of life in older persons [5].

Anaemia in the older person is often unrecognized due to its nonspecific symptoms of weakness and lethargy, which are often mistaken for the ageing process. Anaemia is associated with increasing age and chronic diseases including kidney diseases and diabetes mellitus and is more common in hospitalized individuals [6]. Gaskell et al. 2008 found an association between anaemia with gender and ethnicity, with African Americans and Asians being more likely to develop anaemia than Caucasians. Different ethnicities have varying cultural or religious beliefs, which in turn influence the choices of food, especially iron-rich food such as meat [7]. A previous Malaysian study on racial variations in anaemia among pregnant mothers reported that those of Indian ethnicity were found to have a higher prevalence of anaemia compared to other ethnic groups in Malaysia. Factors associated with anaemia were not identified in this study [8].

The relationship between ethnicity and anaemia has yet to be determined within the Malaysian population. Malaysia is a multiethnic nation, where its population is comprised of three major Asian ethnic groups with a large, heterogeneous indigenous population. This study aims at identifying the potential differences in susceptibility to anaemia among the three major ethnic groups in Malaysia. In addition, using available demographic and medical information, we aimed to identify potential mediators of these differences. An understanding of ethnic differences will facilitate further understanding of the potential influences of cultural practices, dietary factors, and genetic differences on anaemia in older adults.

## 2. Methods

This study was conducted using data from the National Health and Morbidity Survey (NHMS) 2015, a nationwide, cross-sectional survey evaluating the overall health status, health needs, and health expenditure in the Malaysian population. It is a household survey conducted by the Institute for Public Health, Ministry of Health Malaysia in 4 yearly cycles, with the main aim of monitoring noncommunicable diseases and their risk factor burden. Data collection was carried out from March 2015 until May 2015.

Ethical approval for this study was obtained from the Medical Research and Ethics Committee (MREC), Ministry of Health, Malaysia. Written consent was obtained from all respondents. The methodology of this study has been described in the technical report of the NHMS 2015 [9]. Anaemia was included as a topic together with other common health problems among the adult population.

### 2.1. Population and Sampling

The geographical location was stratified into 15 states to ensure national representativeness during the sampling procedure. Multistage random selection was conducted based on enumeration blocks and living quarters provided by the Department of Statistics, Malaysia. For the purpose of this study, only data obtained from individuals aged 60 years and above were included.

### 2.2. Sociodemographic and Clinical Characteristics

The sociodemographic variables of age, ethnicity, education level, marital status, and household income quintiles were included in this study. Respondents were also asked whether they had been admitted to hospital over the past 12 months or received any outpatient treatment over the previous 2 weeks.

Capillary blood sample was tested for haemoglobin level using the HemoCue haemoglobinometer (HemoCue Hb 201+ System, Angelhom, Sweden) [10, 11]. Anaemia was defined as blood haemoglobin levels below 12 g/dL for older women and below 13 g/dL for older men [1]. Glucose levels were tested using Cardiochek PA. These point-of-care procedures were carried out by trained health staff. The presence of diabetes was determined based on a fasting blood glucose measurement of 6.1 mmol/l and above or random blood glucose measurement of 11.1 mmol/l and above. Respondents who were previously diagnosed as having diabetes mellitus by a physician or assistant medical officer would report themselves as “known diabetes.”

### 2.3. Statistical Analysis

IBM SPSS Statistics for Windows, Version 21.0 software, was used for data analysis. Descriptive analysis was conducted to determine the relation between the sociodemographic distribution and anaemia according to the three main ethnic groups: Malays, Chinese, Indians, and a residual “Others” category (which included all Malaysian citizens from indigenous populations). Categorical data were compared with the Chi-squared test while age, which was a continuous variable, was compared using the one-way ANOVA test. Finally, all variables with a *p* value of 0.20 and above were included in a multivariate logistic regression model. The first model was adjusted with all the sociodemographic variables after adjustment for age. The second model identified the relationship between ethnicity and age, diabetes status, and hospital admission. The findings were presented as adjusted odds ratios (aORs) with 95% confidence intervals (CI), and a *p* value < 0.05 was considered significant.

For the sake of both clarity and brevity, this paper will henceforth refer to Malaysian citizens of Malay, Chinese, and Indian ethnicities as simply Malays, Chinese, and Indians, respectively. Malaysian citizens of other ethnicities will be referred to as “Others.”

## 3. Results

The overall response rate for the survey was 86.4%. Out of 3794 older Malaysian citizens, 3556 (93.7%) consented to anaemia screening. The mean age was 68 (±7) years. The respondents consisted of 64.0% Malays, 21.6% Chinese, 6.1% Indians, and 8.3% Others. Gender proportions of the respondents were similar. The majority of the Chinese and Indians lived in urban areas while Malays and others were equally distributed between urban and rural areas. Out of the total population, 11.2% had hospital admissions in the past 12 months while 13.4% had outpatient attendances. Diabetes was present in 38.1% and was most common among the ethnic Indians.  Table 1 summarizes the sociodemographic characteristics of the population according to the four groups.

**Figure 1:**
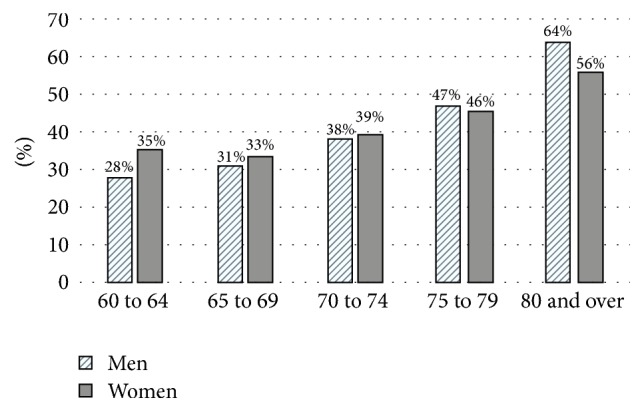
Bar chart comparing prevalence of anaemia by fiver-year age groups according to gender.

Table 2 demonstrates the prevalence of anaemia by sociodemography among the elderly population. The mean (±standard deviation) for haemoglobin levels for the ethnic Malays was 12.8 (±1.8) g/dl, the ethnic Chinese 13.0 (±1.8) g/dl, and the ethnic Indians 12.5 (±1.8) g/dl. The mean haemoglobin for the overall population was 12.8 (±1.8) g/dl. The overall prevalence of anaemia among all older persons was 35.3% (95% CI: 33.15, 37.45). The prevalence of anaemia for Malays, Chinese, Indians, and Others was 36.9 (95% CI: 34.0, 39.9), 31.1% (95% CI: 27.4, 35.1), 42.1% (95% CI: 33.9, 50.1), and 36.3% (95% CI: 30.2, 43.0), respectively. Univariate analyses revealed significant ethnic differences in the prevalence of anaemia. In addition, the sociodemographic factors including age, level of education, marital status, hospital admission in the previous 12 months, and diabetes all differed significantly. The prevalence of anaemia increases from 28% among men in the 60 to 64 years' age group to 64% in men aged 80 years and over. In comparison, 35% of women aged 60–64 years had anaemia while 54% of women aged 80 years and over were anaemic (Figure 1).

Multivariate analyses revealed that following adjustment for age differences, Malays were significantly more likely to be anaemic than Chinese respondents (adjusted odds ratio, aOR = 1.25; 95% CI = 1.04 to 1.49). Indians were also significantly more likely to be anaemic than Chinese respondents after age adjustment (aOR = 1.72; 95% CI = 1.26 to 2.34) (Model 1). In Model 2, following additional adjustment for hospitalization and diabetes mellitus, the Malays and Indians remained more likely to be anaemic than the Chinese (Table 3).

## 4. Discussion

Utilizing data from a nationally representative cross-sectional survey, the elderly Malays and Indians were significantly more likely to have anaemia according to international criteria than the elderly Chinese. This study is continuation from the previous published article regarding anaemia in Malaysian adult population [12].

This was the first study to report ethnic differences in the prevalence of anaemia in an Asian population. Racial differences in anaemia prevalence have been reported in high income general populations or younger populations [13]. A previous study conducted in Singapore had included ethnicity as a variable in their study evaluating anaemia in patients with chronic kidney disease but found no ethnic differences in the prevalence of anaemia among the Chinese, Indian, and Malay populations [14]. However, this study was conducted among hospital patients and in a comparatively smaller population. A recent report on anaemia from the National Health and Nutritional Examination Survey (NHANES) in the United States revealed a much higher prevalence of anaemia among non-Hispanic blacks [15]. Anaemia in older adults, therefore, remains poorly characterized, with much more work being required before effective measures can be considered for this condition, which is likely to pose an increasing burden on society with the rapidly ageing population worldwide.

The prevalence of anaemia in the different ethnic groups in our older population was comparatively higher than that reported in the published literature. A previous systematic review had suggested an overall prevalence of 12% among community-dwelling older adults [6]. The prevalence of 31% to 46% among the different ethnic groups in Malaysia appears to be many folds higher than that reported in studies involving other older populations [16, 17]. The apparently higher prevalence of anaemia in our older population is therefore likely to reflect the higher prevalence of comorbidities especially diabetes mellitus and chronic kidney disease in our older population [9, 18]. In addition, the Asian ethnic groups are also far more likely to have inherited haemoglobinopathies such as thalassaemia minor which may escape clinical detection throughout life and with no known adverse effects on life expectancy [19]. The exact contribution of thalassaemia traits to anaemia in our population is yet to be established.

The higher prevalence of anaemia among our older Indian population is supported by the findings of a study on anaemia prevalence conducted within the Indian subcontinent [20], which utilized the hospital database at a single centre. Their estimated prevalence of over 50% for women of all ages and men aged over 60 years is, however, not comparable to that of our community-based sample. Normocytic anaemia was commonest among their older population suggesting that anaemia could be due to causes other than iron deficiency in their older population. Nevertheless, iron deficiency anaemia has been previously found to be associated with Indian ethnicity as many Hindu devotees are traditionally vegetarians [21, 22]. The bioavailability of vitamin B12 is known to be poor in the traditional Indian vegetarian diet [23].

In terms of nutritional deficiency, in addition to cultural beliefs and religious prohibitions, socioeconomic factors also contribute to nutritional intake as a high socioeconomic status is associated with food diversity and a healthier diet [24]. In general, anaemia is patterned by socioeconomic factors especially by household income whereby those living in the lowest wealth quintiles were found to have 25% higher than among those from the highest quintile [25]. A study done in China reported that a relatively lower risk of anaemia was associated with greater health awareness and a higher household income status [26]. A Singaporean study observed a higher risk of developing iron deficiency anaemia among non-Chinese ethnicities [27]. The aetiology underlying the ethnic differences in anaemia in our population could not be established in this cross-sectional survey. Previous reports have suggested that anaemia in older adults are more likely to occur due to chronic illness than nutritional deficiencies [28]. Following adjustment for hospitalization and the presence of diabetes in our study, however, the ethnic differences in anaemia status remained statistically significant. There is an established relationship between B12 deficiency and diabetes mellitus [27]. In addition, we consider hospitalization as a marker of frailty and medical illness and diabetes as an indicator of chronic disease [29]. Anaemia may also be more likely among those with diabetes due to the increased risk of renal disease [30, 31]. Both Malay and Indian ethnicities were strong independent risk factors for anaemia after the above adjustments, suggesting that the increased risk of anaemia among Indians is not explained by hospitalization nor diabetes. In a previous study involving diabetics in California, the presence of chronic disease accounted for one-third of cases of anaemia and ethnicity remained an independent predictor for anaemia in their diabetic population, for which underlying factors were yet to be established [32]. The absence of any mediators of anaemia in our study population therefore brings into question the likelihood of genetic predilection for anaemia. However, the prevalence of the commonest inherited blood dyscrasia among Asians, beta-thalassemia, has been evaluated among large populations in both China and India with similar prevalence of beta-thalassaemia status of around 3% [33, 34].

As this was a cross-sectional survey, causative relationships could not be established. Haemoglobin in this study was based on point-of-care screening which may lead to a falsely high haemoglobin result by 0.1 g/dL [35]. However, point-of-care screening provides fast and mostly valid results and was felt to be the most appropriate choice to allow for measurement of haemoglobin in remote settings [36]. As a consequence of our choice of measurement tool, there were no associated measurements on cell counts, mean corpuscular volume, haematocrit, or iron studies available. Therefore, it was not possible to determine the type of anaemia in our study population. In addition, as anaemia was assessed as part of a larger national health survey, it was not possible to also accurately assess coexisting medical illness, renal function, or use of medications in our study population. The low percentage of Indian in our population may reduce the accuracy of detection of anaemia in our population. Future studies should now evaluate further the rationale behind the increased risk of anaemia among the older Indians, the relatively higher prevalence of anaemia overall among our older population compared to other population studies, and aim to identify strategies to lower the burden of anaemia among our population, possibly through appropriate chronic disease management. To improve the characterization of anaemia in the ethnic Indians, a stratified sampling method should also be considered.

## 5. Conclusions

Our cross-sectional survey conducted in a multiethnic Asian population has found that both Indian and Malay ethnicities were independent predictors of the presence of anaemia. The differences in prevalence of anaemia in both ethnic Malays and Indians compared to the ethnic Chinese remain significant after adjustment for presence of diabetes and hospitalization in the past 12 months. Our study has identified a high prevalence of anaemia among all ethnic groups, as well as potential modifiable factors to reduce the burden of anaemia among our older population. Further studies should attempt to identify the underlying causes of this common condition among our older population.

## Figures and Tables

**Table 1:** Sociodemographic profiles of the respondents 4 major ethnicities in Malaysia.

Variables	Overall (*n* = 3794)	Malay (*n* = 2,429)	Chinese (*n* = 818)	Indian (*n* = 232)	Others (*n* = 315)
	*n* (%)	*n* (%)	*n* (%)	*n* (%)	*n* (%)
*Age Mean (SD)*	68.56 (7.11)	68.30 (6.89)	69.17 (7.39)	68.80 (7.50)	68.82 (7.65)
*Gender*					
Male	1773 (48.97)	1115 (47.55)	401 (51.96)	98 (44.00)	159 (48.63)
Female	2021 (51.03)	1314 (52.45)	417 (48.04)	134 (56.00)	156 (51.37)
*Level of Education*					
No Formal Education	799 (21.39)	432 (18.60)	150 (15.85)	48 (16.35)	169 (56.66)
Primary	1959 (47.59)	1364 (51.29)	403 (49.54)	92 (39.11)	100 (29.05)
Secondary and higher	1025 (31.02)	627 (30.11)	261 (34.62)	91 (44.54)	46 (14.29)
*Marital Status*					
Married	2558 (68.26)	1641 (67.30)	557 (70.03)	136 (63.77)	224 (69.56)
Currently Not Married	1234 (31.74)	787 (32.70)	260 (29.97)	96 (36.23)	91 (30.44)
*Household Income *					
Q1 (RM0–RM300)	1224 (31.01)	773 (28.13)	259 (32.13)	61 (25.60)	131 (44.24)
Q2 (RM301–RM1100)	879 (20.84)	628 (25.44)	143 (15.29)	44 (17.27)	64 (20.59)
Q3 (RM1101–RM2009)	674 (16.66)	460 (18.99)	116 (15.32)	50 (13.89)	48 (12.05)
Q4 (RM2010–RM4000)	505 (14.70)	293 (13.69)	135 (16.42)	30 (11.70)	47 (15.49)
Q5 (RM4001–RM205690)	512 (16.79)	275 (13.75)	165 (20.84)	47 (31.55)	25 (7.63)
*Strata*					
Urban	1850 (71.99)	922 (59.53)	655 (93.78)	176 (94.84)	97 (40.99)
Rural	1944 (28.01)	1507 (40.47)	163 (6.22)	56 (5.16)	218 (59.01)
*Hospitalization*					
Yes	402 (11.15)	270 (12.84)	69 (9.10)	22 (8.51)	41 (11.93)
No	3388 (88.85)	2158 (87.16)	747 (90.90)	210 (91.49)	273 (88.07)
*Out Patient Attendance*					
Yes	504 (13.38)	317 (13.71)	95 (11.06)	30 (13.24)	62 (19.88)
No	3286 (86.62)	2111 (86.29)	721 (88.94)	202 (86.76)	252 (80.12)
*Diabetes Mellitus*					
Yes	1463 (38.05)	963 (40.88)	274 (34.00)	136 (56.02)	90 (26.99)
No	2327 (61.95)	1465 (59.12)	541 (66.00)	96 (43.98)	225 (73.01)

**Table 2:** Prevalence of anaemia by socio-demo characteristics among elderly (*n* = 3566).

	Anaemia								
Variables	Yes				No				
	*n*	%	95% CI		*n*	%	95% CI		*p* value
			lower	upper			lower	upper	
*Malaysia*	1311	35.27	33.15	37.45	2245	64.73	62.55	66.85	
*Age *									
Mean, (SD)	69.98 (7.89)	-	-	-	67.86 (6.70)	-	-	-	<0.001
*Ethnicity*									
Malays	852	36.92	34.03	39.91	1453	63.08	60.09	65.97	0.035
Chinese	238	31.10	27.37	35.10	483	68.90	64.90	72.63	
Indians	101	42.07	33.91	50.69	121	57.93	49.31	66.09	
Others	120	36.34	30.17	42.99	188	63.66	57.01	69.83	
*Gender*									
Male	581	32.85	30.06	35.77	1071	67.15	64.23	69.94	0.025
Female	730	37.57	34.54	40.70	1174	62.43	59.30	65.46	
*Level of Education*									
No Formal Education	315	40.21	35.86	44.72	436	59.79	55.28	64.14	0.010
Primary	688	35.75	32.77	38.85	1159	64.25	61.15	67.23	
Secondary and higher	306	31.13	27.37	35.16	649	68.87	64.84	72.63	
*Marital Status*									
Married	832	33.36	30.91	35.92	1575	66.64	64.08	69.09	0.010
Currently Not Married	479	39.38	35.48	43.43	670	60.62	56.57	64.52	
*HH Income Group*									
Q1 (RM0–RM300)	446	34.69	31.23	38.32	696	65.31	61.68	68.77	0.715
Q2 (RM301–RM1100)	296	35.13	30.77	39.75	534	64.87	60.25	69.23	
Q3 (RM1101–RM2009)	236	17.43	14.19	21.22	398	63.09	56.99	68.80	
Q4 (RM2010–RM4000)	163	13.75	11.20	16.78	313	67.57	61.82	72.84	
Q5 (RM4001–RM205690)	170	37.44	32.21	42.98	304	15.95	13.66	18.54	
*Strata*									
Urban	620	35.26	32.56	38.07	1080	64.74	61.93	67.44	0.996
Rural	691	35.28	32.29	38.38	1165	64.72	61.62	67.71	
*Hospitalization*									
Yes	188	16.96	13.61	20.94	189	8.23	6.67	10.12	<0.001
No	1122	32.96	30.86	35.13	2056	67.04	64.87	69.14	
*Out Patient Attendance*									
Yes	188	37.30	31.91	43.02	292	62.70	56.98	68.09	0.437
No	1122	34.88	32.55	37.28	1953	65.12	62.72	67.45	
*Diabetes Mellitus*									
Yes	569	38.64	35.21	42.20	835	61.36	57.80	64.79	0.011
No	742	33.09	30.49	35.79	1410	66.91	64.21	69.51	

**Table 3:** Multivariate analysis for factors associated with anaemia in Malaysian older adults in relation to ethnicity.

	Wald Test	df	Adjusted OR	95% CI		*p* value
				Lower	Upper	
Model 1^a^						
*Ethnicity*	13.06	3				0.005
Chinese	-	-	1.00	-	-	-
Malay	5.76	1	1.25	1.04	1.49	0.016^*∗*^
Indians	11.53	1	1.72	1.26	2.34	0.001^*∗∗*^
Others	4.03	1	1.33	1.01	1.76	0.045^*∗*^
*Age*	70.98	1	1.04	1.03	1.05	<0.001^*∗∗∗*^
Model 2^b^						
*Ethnicity*	11.31	3				0.010^*∗*^
Chinese	-	-	1.00	-	-	-
Malay	4.71	1	1.22	1.02	1.46	0.030^*∗*^
Indians	10.00	1	1.66	1.21	2.27	0.002^*∗∗*^
Others	3.60	1	1.32	0.99	1.75	0.058
*Age*	80.82	1	1.05	1.04	1.06	<0.001^*∗∗∗*^
*Hospitalization*						
Yes	20.91	1	1.67	1.34	2.08	<0.001^*∗∗∗*^
No	-	-	1.00	-	-	-
*Diabetes Mellitus*						
Yes	10.29	1	1.26	1.10	1.46	0.001^*∗∗*^
No	-	-	1.00	-	-	-

^a^Adjusted for ethnicity, age, sex, strata, household income quintile, education and marital status; ^b^Adjusted for ethnicity, age, diabetes, hospitalization, outpatient attendance; ^*∗*^*p* < 0.05; ^*∗∗*^*p* < 0.005; ^*∗∗∗*^*p* < 0.0005.
